# Knowledge Management and Digital Innovation in Healthcare: A Bibliometric Analysis

**DOI:** 10.3390/healthcare12242525

**Published:** 2024-12-13

**Authors:** Angelos I. Stoumpos, Michael A. Talias, Christos Ntais, Fotis Kitsios, Mihajlo Jakovljevic

**Affiliations:** 1 Healthcare Management Program, School of Economics and Management, Open University of Cyprus, 2220 Nicosia, Cyprus; angelos.stoumpos1@st.ouc.ac.cy (A.I.S.); christos.ntais@st.ouc.ac.cy (C.N.); 2 Department of Applied Informatics, University of Macedonia, 54636 Thessaloniki, Greece; kitsios@uom.gr; 3 Institute of Advanced Manufacturing Technologies, Peter the Great St. Petersburg Polytechnic University, 195251 St Petersburg, Russia; jakovljevicm@medf.kg.ac.rs; 4 Institute of Comparative Economic Studies, Hosei University, Tokyo 102-8160, Japan; 5 Department of Global Health Economics and Policy, University of Kragujevac, 34000 Kragujevac, Serbia

**Keywords:** digital innovation, health systems, healthcare, knowledge management, bibliometric analysis

## Abstract

Background/Objectives: In recent years, knowledge management and digital innovation have become pivotal in transforming healthcare systems, driving efficiency and enhancing patient outcomes. This study presents a bibliometric analysis of research trends at the intersection of knowledge management and digital innovation in healthcare, examining the scope, impact, and evolution of scholarly work in this domain. Methods: Using a comprehensive dataset from the Scopus database, we analyzed 419 publications from 1985–2023 to identify influential authors, journal collaborations, and emerging topics and methodologies in the field. Results: Our findings reveal a significant increase in research interest, highlighting themes such as data-driven healthcare, artificial intelligence in clinical decision support, and knowledge-sharing platforms’ role in improving healthcare delivery. The analysis also underscores the growing importance of interdisciplinary collaboration between healthcare providers, technologists, and policy-makers. Conclusions: By mapping the intellectual structure of knowledge management and digital innovation in healthcare, this study provides valuable insights for academics, practitioners, and policy-makers seeking to harness knowledge management practices and digital technologies to foster innovation and resilience in healthcare systems.

## 1. Introduction

The healthcare sector has witnessed a rapid transformation driven by digital innovation, encompassing technology-driven changes that enhance healthcare delivery, improve patient outcomes, and streamline operational efficiency. Digital innovation in healthcare includes advances such as artificial intelligence (AI), telemedicine, electronic health records, and wearable health devices, which have reshaped how healthcare services are provided and accessed [1,2,3]. This shift occurs within complex health systems—the interconnected organizations, resources, and policies designed to deliver healthcare services and improve public health outcomes [4,5,6]. Health systems are the backbone of healthcare delivery, ensuring accessible, equitable, and efficient healthcare for populations [7]. To improve healthcare delivery, digital innovation refers to adopting advanced technologies, including AI, telemedicine, wearable devices, and electronic health records (EHRs). Digital solutions empower providers to streamline workflows, enhance patient engagement, and guide data-driven decision-making. Key areas of digital innovation in healthcare include: (i) AI and machine learning—AI enables predictive analytics and real-time diagnostics, from early disease detection to personalized treatment recommendations. Machine learning algorithms can process vast datasets to identify patterns, flagging potential health issues before symptoms appear; (ii) telemedicine and remote monitoring: telemedicine platforms have allowed providers to extend care to remote areas, reduce in-clinic wait times, and offer consultations across geographic barriers [8,9,10]. Remote monitoring, through wearables or smart devices, provides continuous patient data, allowing for timely intervention; (iii) EHRs—EHRs offer a centralized, digital repository of patient records, enhancing collaboration among providers, improving accuracy, and reducing redundant tests or procedures; (iv) blockchain technology: blockchain provides a secure, immutable record of patient information, safeguarding data privacy and enabling transparent data sharing between trusted parties.

Closely related is the concept of digital transformation, defined as: “a fundamental change process enabled by digital technologies that aim to bring radical improvement and innovation to an entity (e.g., an organization, a business network, an industry, or society) to create value for its stakeholders by strategically leveraging its essential resources and capabilities” [11]. Digital transformation extends beyond isolated innovations to encompass the strategic, organization-wide integration of digital technologies. Digital transformation involves adopting specific innovations and fundamentally reshaping processes, business models, and organizational cultures to leverage digital advancements fully. In healthcare, digital transformation includes reconfiguring care delivery models and patient engagement strategies, leading to more personalized, efficient, and accessible care. Together, these concepts, digital innovation and digital transformation, frame our study’s focus as we examine the evolving role of knowledge management in supporting digital advancements across healthcare settings.

Integrating digital solutions requires effective knowledge management, which involves systematically organizing, sharing, and analyzing medical data and expertise to enhance clinical and operational decision-making. Healthcare organizations must manage knowledge to facilitate continuity of care, uphold high standards, and ensure the latest medical advances and protocols are available to clinicians. Effective knowledge management is essential for: (i) clinical decision support—by organizing patient histories, lab results, and evidence-based guidelines, knowledge management aids clinicians in making accurate, timely diagnoses and treatment plans; (ii) continuous improvement—knowledge management allows healthcare organizations to learn from past successes and challenges, optimizing their approach to patient care and administration; (iii) interdisciplinary collaboration—knowledge management systems promote seamless information sharing between departments, enabling integrated and patient-centered care.

Integrating knowledge management and digital innovation reshapes healthcare, promising enhanced patient outcomes, operational efficiency, and accelerated decision-making [12]. This study addresses that gap by mapping the intellectual landscape of knowledge management and digital innovation in healthcare from 1985 to 2023, providing a critical resource for researchers, practitioners, and policymakers. Our analysis highlights prominent research areas, uncovers collaborative networks, and identifies future directions for research and practice. By consolidating the knowledge base, this study contributes to advancing the field and guiding future efforts in leveraging knowledge management and digital technologies to improve healthcare systems. Our analyses aim to provide a comprehensive overview of critical topics, influential researchers, and institutional collaborations in knowledge management and digital innovation in healthcare. This bibliometric mapping reveals research trends, significant publication sources, and emerging themes, ultimately guiding future research directions and supporting evidence-based innovation in healthcare management.

## 2. Integrating Knowledge Management and Digital Innovation in Modern Healthcare

Digital innovation and knowledge management are symbiotic in healthcare. Digital tools provide platforms for capturing, storing, and analyzing knowledge. Integrating knowledge management with digital solutions like AI or telemedicine allows healthcare providers to leverage data for immediate care and long-term learning.

### 2.1. Clinical Decision Support Systems (CDSS)

CDSS combines knowledge management with AI algorithms, providing healthcare professionals with real-time insights and alerts based on current patient data. For instance, a CDSS may alert a physician if a patient’s symptoms match a specific condition or if their medication dosage requires adjustment, facilitating evidence-based care.

### 2.2. Personalized Patient Care

With wearable technology and data analytics, personalized medicine is now more accessible. Physicians can tailor treatment plans based on patient-specific factors, from genetic information to lifestyle habits. Knowledge management enables providers to retain and apply these insights across similar cases, advancing personalized care practices industry-wide.

### 2.3. Improved Operational Efficiency

Knowledge management and digital tools streamline hospital administration by automating scheduling, billing, and inventory management. Predictive analytics can forecast resource demand, reducing patient flow bottlenecks and ensuring efficient use of facilities and staff.

### 2.4. Enhanced Research and Development

Knowledge repositories and data-sharing networks facilitate research collaboration and accelerate the development of new treatments. For example, knowledge management enables research institutions to pool anonymized patient data, enhancing the understanding of disease progression and treatment outcomes and ultimately driving innovation in drug discovery.

## 3. Methodology

We followed a systematic approach to conduct a bibliometric analysis of healthcare knowledge management and digital innovation. Our process involved the following stages:

### 3.1. Data Collection

We sourced data from the Scopus database, which were collected in November 2024. Scopus is recognized for its extensive coverage of peer-reviewed literature and rigorous indexing standards [1]. We limited our search to articles published from 1985 to 2023 to capture foundational studies and recent advancements in the field. This extended timeframe allowed us to observe trends over time, offering insights into the evolution of digital innovation in healthcare.

### 3.2. Inclusion Criteria

We focused on articles on healthcare, knowledge management, and digital innovation and filtered results based on relevance to the study’s aims. By using specific keywords (“knowledge management”, “digital innovation”, “healthcare systems”, “digital health”, etc.), we narrowed our search to articles specifically focused on healthcare settings and management applications to ensure relevance and quality of insights.

### 3.3. Data Extraction and Selection

After identifying relevant articles, we extracted data from titles, abstracts, and keyword sections. This targeted approach allowed us to focus on studies directly contributing to the intersections of our primary research interests.

### 3.4. Data Processing and Analysis

The collected data were processed using the bibliometric tool VOSviewer v.1.6.20, a software developed by Van Eck and Waltman, that enables advanced analyses of citation networks, co-authorship patterns, keyword co-occurrence, and research trends [13]. We chose this tool for its capabilities in visualizing complex networks and identifying influential research themes and collaborations.

Network analysis is an increasingly popular tool for analyzing complex relationships between sectors and industries. Measuring all aspects of networks has made network analysis a central tool in analyzing bibliometric data [14].

### 3.5. Citation Analysis

This approach helped us identify the most cited articles and journals, highlighting seminal works that have shaped the field [15].

### 3.6. Co-Authorship and Collaboration Networks

By mapping co-authorship networks, we observed influential authors and institutions and international collaboration trends in healthcare digital innovation research.

### 3.7. Keyword Co-Occurrence Analysis

This method enabled us to identify frequently discussed topics and emerging trends, providing insights into the most relevant themes within the field.

## 4. Results

Four hundred and nineteen articles were retrieved, with a growing trend in the volume of publications over the years. The most relevant keywords are listed in Table 1.

**Figure 1:**
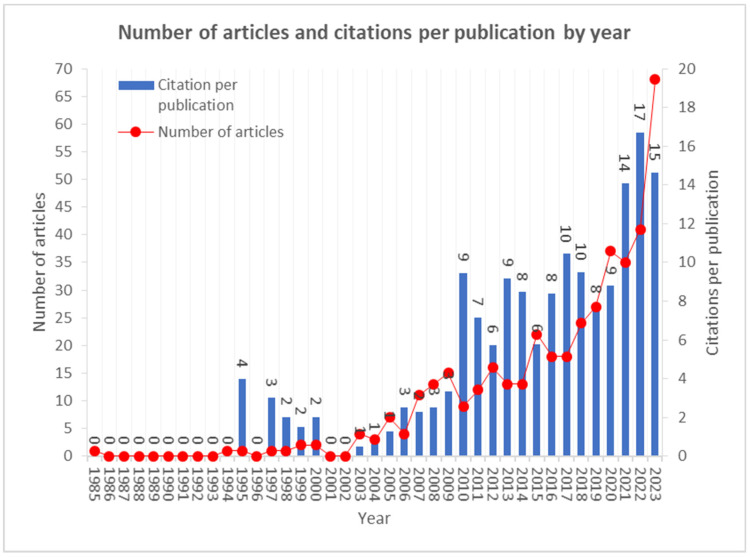
Number of articles and citations per publication by year.

The first studies date back to 1985, though no articles were published in 1986–1994, 1996, and 2001–2002. On the other hand, there has been a gradual increase in publications on managing digital health knowledge since 2004. Figure 1 presents the number of published articles per year and the number of citations per year. From 2005 onwards, we observe an increasing trend regarding the number of citations per year. However, the increase in articles per year shows fluctuations in citations, which increase exponentially per year.

**Figure 2:**
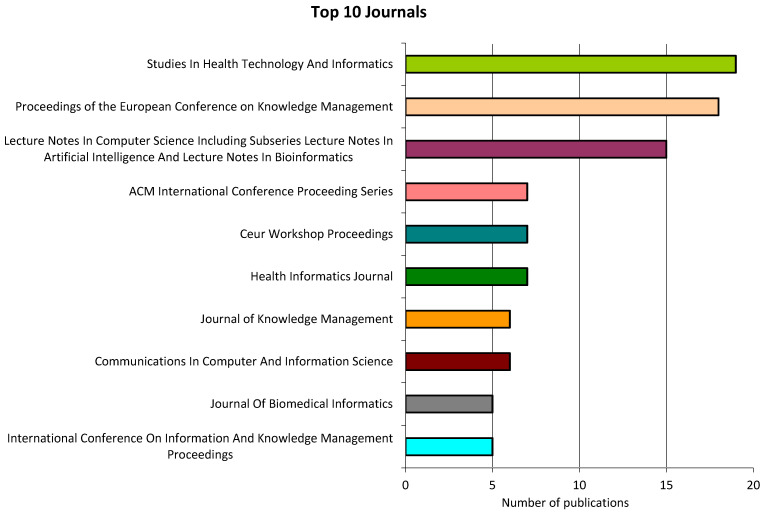
Top 10 journals.

Figure 2 shows the journals with the most publications. 

Table 2 lists the top seven organizations publishing the most articles. The Consiglio Nazionale delle Ricerche of Italy has published seven. The University of Toronto in Canada has published six, followed by the RMIT University of Australia and the University of Washington in the USA, each of which has published four.

**Figure 3:**
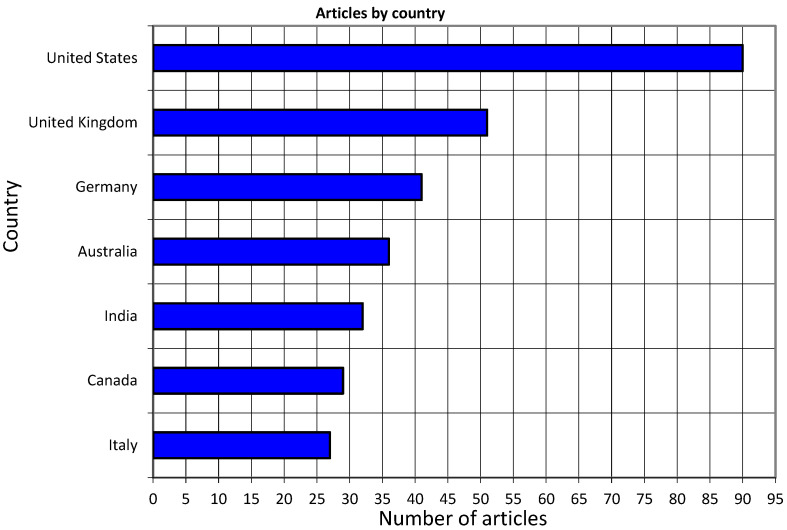
Top seven countries by number of articles.

Figure 3 presents the number of publications by country. The United States of America is at the top, with 90 publications. The United Kingdom follows with 51 articles and Germany with 41 articles. Australia has published 36 articles, India follows with 32, Canada has published 32 articles, and Italy follows with 27.

**Figure 4:**
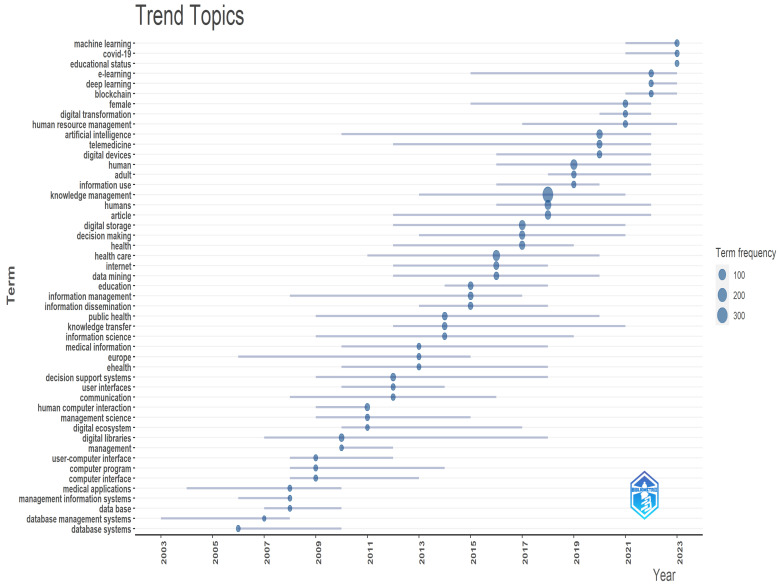
Topic trend analysis.

Figure 4 shows the topic trend graph displays the frequency of utilisation for different keywords.

This is an overview of the topics that are becoming important in knowledge management, innovation, and healthcare. This figure helps to show that topics are gaining significance in the field each year.

**Figure 5:**
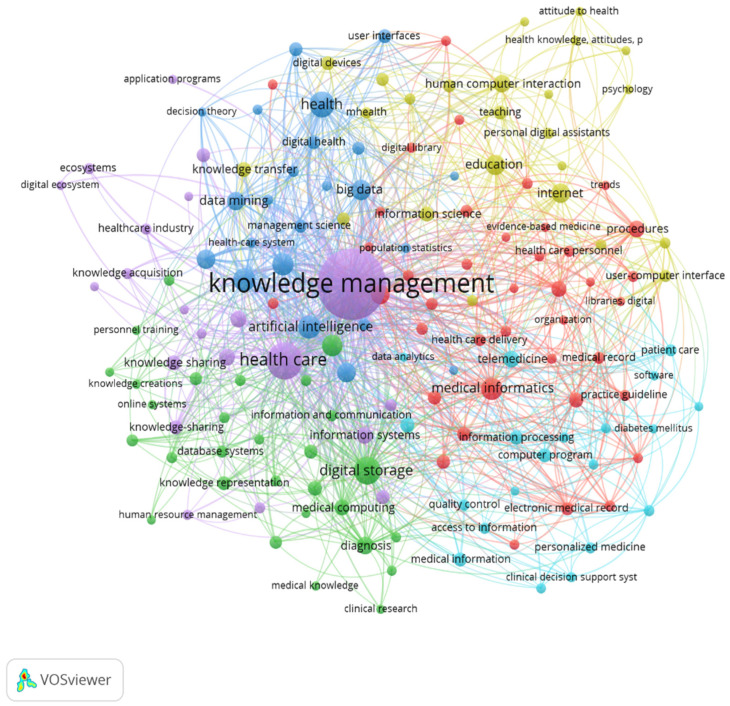
Network visualization of author keywords.

Figure 5 represents a grid focused on keyword reproduction in the literature on the general dimensions of digital knowledge management in health. It shows that knowledge management is linked to the health and digital sectors.

**Figure 6:**
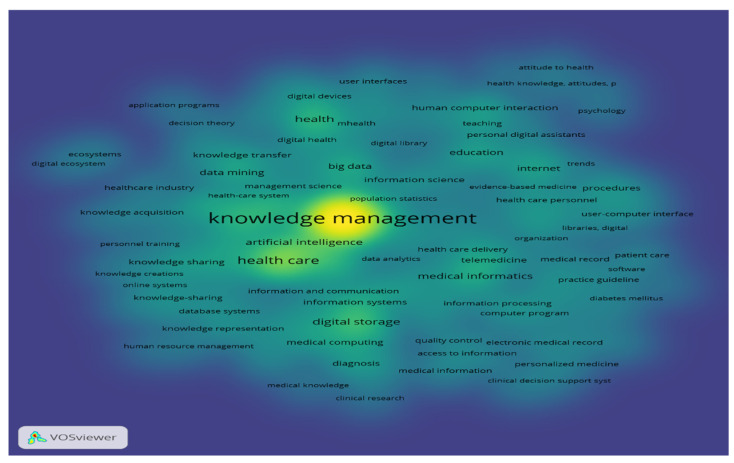
Density Visualization Map of Keywords.

Figure 6 shows the density visualization map of keywords, showing that the most prevalent nodes are knowledge management, health care, health, internet, and digital storage.

**Figure 7:**
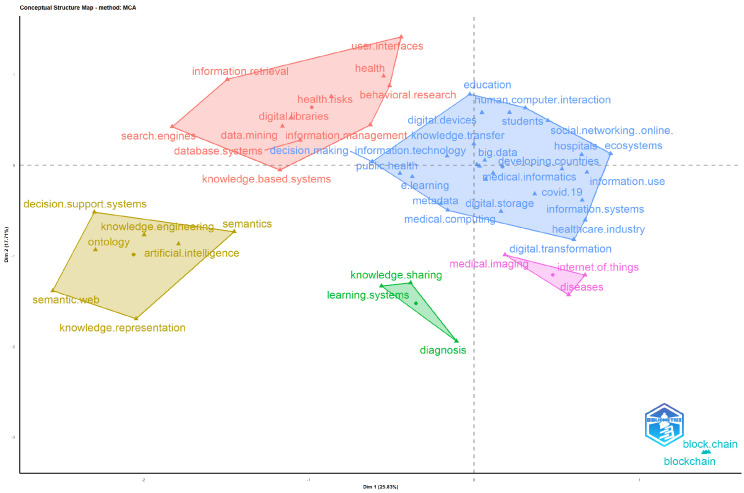
Multiple Correspondence Analysis (MCA).

Figure 7 illustrates the results of a multiple correspondence analysis (MCA) of the keywords used. The map shows six clusters, each encompassing keywords related to the knowledge management process of digital healthcare. The red cluster refers to the database systems and information retrieval during the knowledge process. The yellow cluster refers to the decision support systems procedures related to knowledge engineering and knowledge representation through artificial intelligence. The blue cluster represents digital health procedures and how these are related to human-technology interaction, such as digital storage, human-computer interaction, and medical computing. The green cluster refers to learning systems and diagnostics procedures through knowledge sharing. The blockchain is the smallest cluster (bottom right).

## 5. Discussion

Our results indicate that the “innovation” node is directly linked to the “knowledge management”, “health”, and “digital healthcare” nodes. In other words, a two-way relationship is crucial for the health sector. This highlights the importance of health knowledge and digitalization in health. Digital repositories of knowledge can make a decisive contribution to both the evolution of a patient’s health and to research and development. In addition, they contribute to innovative applications and solutions in the healthcare field while saving time, money, and staff. Due to their structure and nature, digital knowledge repositories can be integrated into artificial intelligence or cloud computing systems, upgrading the role and function of these systems and the patient care provided. In addition, a digital health knowledge repository is constantly being enriched by evidence yielded by domestic and international research efforts through thematic networks of innovation and open consultation [16].

One of the things that promotes innovation is knowledge sharing. Only through knowledge exchange is innovation possible [17]. Collaboration is an efficient and beneficial way to acquire information and skills for successful innovation. Regarding creation, knowledge exchange refers to sharing skills and knowledge to produce or enhance valuable goods and services. Knowledge transfer is a critical process that enables organizations to share expertise, skills, and experience across teams, projects, and departments. Based on a meta-analysis, Mesmer-Magnus and DeChurch concluded that knowledge sharing can forecast team performance [18]. Subsequently, ignorance is the primary barrier to innovation. Darroch and McNaughton [19] asserted that a company promoting knowledge exchange is likelier to foster creative thinking and new ideas. We could extrapolate their viewpoint regarding hospitals. Furthermore, Belso and Diez found that businesses become more innovative when participating in knowledge networks [20].

Several studies have examined the connection between innovation and knowledge sharing without, however, having considered their role in the development and application of digital health. Numerous researchers have emphasized the need to investigate knowledge exchange and innovation. Tamer Cavusgil et al. [21] concluded that an organization’s capacity for innovation increases with the amount of tacit knowledge transferred. The same rule could apply to hospitals. Sharing tacit information is crucial for creativity. One way of turning implicit knowledge into explicit knowledge is through knowledge sharing. Knowledge of both kinds can be used to spark innovation.

Camelo et al. [22] found that knowledge sharing positively affected innovation in a survey of Spanish organizations. This was also pointed out by Podrug et al. [23] in Croatian companies, where knowledge sharing increased the capacity for innovation. Taminiau et al. [24] found that informal knowledge sharing is the most fruitful path to innovation. Mura et al. [25] explained how knowledge sharing and innovation are linked. In their study, any health behaviors associated with knowledge sharing positively affect innovation regarding the tendency and ability to promote and implement new ideas. Wang and Hu [26] argued that knowledge sharing mediates collaborative innovation and organizational performance. It has also been shown that knowledge sharing enhances subjective well-being and individual innovation [27]. There is also evidence that knowledge sharing between employees in an industry can drive innovation [28]. On the other hand, Kamasak and Bulutlar [29] found that knowledge sharing within a group influenced exploitative innovation.

## 6. Conclusions

Digital health transformation is an urgent need dictated by the constant and rapid changes and innovations in the IT and healthcare sectors. By adapting to new requirements, health systems acquire new knowledge based on which new medical protocols and tools can be developed to benefit patients and healthcare staff. There is a research gap regarding digital health knowledge management. In addition, the health knowledge already acquired must be manageable. This implies adapting existing health models, theories, and practices to new conditions. New ways of collecting knowledge need to be developed in the health sector, as digital transformation allows health systems to apply new technologies for disseminating knowledge at all levels, along with automated systems often based on artificial intelligence. In addition, proper knowledge management could increase the productivity and efficiency of healthcare personnel. At the same time, patients could be served more efficiently, with greater accuracy in terms of diagnosis and best practices to their benefit.

The main conclusion of this research is the need to promote and manage health knowledge through digital systems, as the current situation cannot meet the requirements related to the daily challenges faced by patients and healthcare staff. In addition, by adopting and using such health innovations, new knowledge can be disseminated faster, more securely, and more cost-effectively, and it can also be stored for future use.

While integrating knowledge management and digital innovation holds immense potential, several challenges persist, including: (i) Data privacy and security: protecting sensitive patient information is critical. As more data are collected and shared, ensuring confidentiality and compliance with regulations such as HIPAA (in the U.S.) or GDPR (in Europe) is paramount. Blockchain and encryption technologies offer solutions, but widespread implementation requires investment and regulatory alignment; (ii) Interoperability: different healthcare organizations often use disparate knowledge management and digital systems, hindering seamless data exchange. Building interoperable systems is necessary to maximize the potential of knowledge management and digital tools across diverse healthcare environments; (iii) Change management and training: integrating digital tools and knowledge management requires significant shifts in organizational culture and workforce skills. Training clinicians and staff on new systems is vital, as is ensuring leadership buy-in to foster an environment open to digital transformation; (iv) Cost and resource constraints: developing, implementing, and maintaining knowledge management and digital innovation systems can be costly, particularly for smaller healthcare providers. Balancing initial investments with long-term benefits is a constant challenge.

Knowledge management and digital innovation are reshaping healthcare by equipping providers with the tools to deliver more efficient, personalized, and data-driven care. As knowledge management and digital systems become increasingly sophisticated, the healthcare industry can expect even more significant improvements in patient outcomes, operational efficiency, and research capabilities. Embracing these strategies will require overcoming data privacy, interoperability, and organizational culture challenges, but the benefits outweigh the costs. By continuing to innovate and invest in knowledge management, healthcare organizations can create a resilient, patient-centered system that meets the evolving needs of modern healthcare.

## 7. Limitations and Further Research

While our bibliometric analysis provides a comprehensive overview of knowledge management and digital innovation in healthcare, certain limitations should be noted. First, our study relied on data from one central database, Scopus, which, although widely regarded for its extensive coverage, may exclude relevant studies indexed in other databases or non-English publications. This choice could limit the generalizability of our findings across diverse regions or languages. Future research could benefit from including additional databases, such as Web of Science (Wos), PubMed, or IEEE Xplore, to capture a broader range of studies and enhance representativeness.

Additionally, our analysis centered on citation counts, co-authorship networks, and keyword co-occurrence to identify key themes, researchers, and collaborations. These bibliometric methods provide valuable insights but may not fully capture the qualitative aspects of digital innovations or the practical impact on healthcare delivery. Future research could incorporate a qualitative review of selected articles to deepen understanding of specific innovations’ impacts and applications.

Finally, our study does not address potential cultural, regulatory, or organizational factors that influence the adoption of digital innovations in healthcare across different countries. Future studies could explore these contextual factors, providing insights into how healthcare systems in various regions approach digital innovation and knowledge management differently.

## Figures and Tables

**Table 1:** Most Relevant Keywords.

Keywords	No of Articles	Keywords-Plus	No of Articles
Knowledge Management	99	Knowledge Management	325
Healthcare	24	Health Care	89
Big Data	18	Human	81
Digital Health	18	Humans	68
Knowledge Sharing	14	Article	56
Ontology	13	Digital storage	55
Artificial Intelligence	11	Artificial Intelligence	48
Cloud Computing	9	Medical Informatics	43
COVID-19	9	Decision Making	37
Digital Transformation	9	Health	36

**Table 2:** Number of Articles per Organization.

Articles per Organization		
Country	Organisation	No. of Articles
Italy	Consiglio Nazionale delle Ricerche	7
Canada	University of Toronto	6
Italy	Università degli Studi di Napoli Federico II	5
Canada	University of Alberta	5
Finland	Tampere University	4
Australia	RMIT University	4
USA	University of Washington	4

## Data Availability

Data are contained within the article.
